# Prevalence of hepatitis C virus infection in Qatar’s resident population based on a national screening campaign

**DOI:** 10.1038/s41598-025-96722-z

**Published:** 2025-04-18

**Authors:** Hamad Eid Al-Romaihi, Rayane El-Khoury, Sayed Himatt, Moutaz F. M. Derbala, Amjad Mohammed Idries, Abid Saeed, Maysa Kamal Abdelmageed, Khalid Hamid Elawad, Merin Alex, Mohamed Sallam, Maha Hammam Al-Shamali, Peter Coyle, Saad Alkaabi, Hiam Chemaitelly, Devendra Bansal, Laith J. Abu-Raddad

**Affiliations:** 1 Department of Health Protection and Communicable Diseases Control, Ministry of Public Health, Doha, Qatar; 2 Infectious Disease Epidemiology Group, Weill Cornell Medicine-Qatar, Cornell University, Qatar Foundation - Education City, P.O. Box 24144, Doha, Qatar; 3 World Health Organization Collaborating Centre for Disease Epidemiology Analytics on HIV/AIDS, Sexually Transmitted Infections, and Viral Hepatitis, Weill Cornell Medicine–Qatar, Cornell University, Foundation – Education City, Doha, Qatar; 4 Eastern Mediterranean Public Health Network, Amman, Jordan; 5 Gastroenterology and Hepatology Department, Hamad Hospital, Doha, Qatar; 6 Department of Preventative Health - Health Protection, Primary Health Care Corporation Head Office, Doha, Qatar; 7 Performance and Evaluation Advisors (PEA) Consultancy, Cairo, Egypt; 8 Hamad Medical Corporation, Doha, Qatar; 9 Department of Biomedical Science, College of Health Sciences, QU Health, Qatar University, Doha, Qatar; 10 Wellcome-Wolfson Institute for Experimental Medicine, Queens University, Belfast, UK; 11 Department of Population Health Sciences, Weill Cornell Medicine, Cornell University, New York, NY USA; 12 Department of Public Health, College of Health Sciences, QU Health, Qatar University, Doha, Qatar; 13 College of Health and Life Sciences, Hamad Bin Khalifa University, Doha, Qatar

**Keywords:** Hepatitis C, Epidemiology

## Abstract

Hepatitis C virus (HCV) infection poses a global health challenge, yet its epidemiology in Qatar remains underexplored. This study estimated HCV antibody (Ab) prevalence in Qatar and examined associated socio-demographic factors. From January 2017 to December 2019, the Ministry of Public Health conducted a national HCV awareness and surveillance campaign. A total of 81,615 individuals, including both Qatari nationals and expatriate residents from 137 countries, underwent HCV antibody testing using the Elecsys Anti-HCV II electrochemiluminescence immunoassay. Probability weights were applied to adjust for age, sex, and nationality, to the testing sample. Logistic regression assessed factors associated with HCV Ab positivity. Among individuals tested, 1149 were positive, 80,299 were negative, and 167 were indeterminate. The estimated HCV Ab prevalence in the study sample was 1.4% (95% CI 1.3–1.5%). The weighted HCV Ab prevalence in the resident population of Qatar was also estimated at 1.4% (95% CI 1.2–1.7%). HCV Ab positivity was strongly associated with age, with higher adjusted odds ratios (AORs) in older individuals. Disparities were noted among certain nationalities, with Bangladeshis and Tunisians having lower odds of infection, while Pakistanis, Egyptians, and Yemenis had higher odds of being infected. Males had a 1.97-fold (95% CI 1.47–2.65) higher odds of being HCV Ab positive compared to females. The considerable HCV Ab prevalence in Qatar underscores the need for targeted interventions, prevention and harm reduction strategies, and expanded testing and treatment programs to advance progress toward the 2030 elimination target.

**Supplementary Information:**

The online version contains supplementary material available at 10.1038/s41598-025-96722-z.

## Introduction

Hepatitis C virus (HCV) infection poses a global health challenge^1^, contributing significantly to morbidity and mortality, particularly through disease progression to fibrosis, cirrhosis, and liver cancer^2–4^. The primary mode of transmission is through exposure to infected blood or bodily fluids, often occurring through needle sharing, unsafe medical procedures, unscreened blood transfusions, and, rarely, through sexual contact or mother-to-child transmission^1,5–7^. According to the Global Burden of Disease Study, viral hepatitis ranks as the seventh leading cause of death worldwide, with HCV infection accounting for half of this burden^1^.

In 2019, the World Health Organization (WHO) estimated a total of 58 million chronic HCV infections worldwide, with 1.5 million new infections occurring that year^6^. In response to this global health challenge, the WHO devised the Global Health Sector Strategy 2022–2030 with the goal of eliminating viral hepatitis as a global health threat^4^. As part of this strategy, the WHO set a target to reduce the incidence rate of HCV infection in all countries to less than 5 cases per 100,000 by the year 2030^4^.

HCV testing and detection typically involve serological assays targeting HCV antibodies, followed by molecular testing for confirmation of current infection^8^. The development of curative pan-genotypic direct-acting antivirals (DAAs), which achieve sustained virological responses of over 95% across all HCV genotypes, represents a groundbreaking advancement in HCV treatment and control^9,10^. The increased accessibility of DAAs, facilitated by generic formulations and substantially reduced prices in resource-limited countries^10,11^, highlights the potential to eliminate HCV infection as a public health threat within a short timeframe^10,12^.

Qatar is a country situated in the Middle East and North Africa (MENA) region, the region that bears the highest burden of HCV infection among all regions^5,13–16^. The first objective of this study was to estimate HCV antibody (Ab) prevalence in the resident population of Qatar and to explore the socio-demographic factors associated with infection. This investigation utilized data from a large HCV national awareness and surveillance campaign that screened over 80,000 individuals for HCV antibodies.

Given that expatriates comprise 89% of Qatar’s 2.8 million population, representing over 150 nationalities drawn primarily for employment amid the country’s rapid economic development over the past two decades^17–20^, the second objective was to estimate HCV Ab prevalence among various expatriate nationalities, particularly those originating from resource-limited countries where data on HCV Ab prevalence are scarce^21–24^.

Ultimately, this study aims to enhance our understanding of HCV epidemiology both locally in Qatar and globally by providing epidemiological insights and contributing meaningful data to inform national initiatives in Qatar and other countries that strive to achieve the global targets for eliminating viral hepatitis. Critically, the findings of this sero-surveillance analysis can guide efforts to expand targeted interventions within Qatar’s diverse resident population of numerous nationalities, improve equitable healthcare access, and optimize existing healthcare infrastructure to accelerate progress toward HCV elimination by 2030.

## Methods

### Study sample, data sources, and HCV antibody testing

This study presents results from HCV Ab testing during a national awareness and surveillance campaign for HCV infection in Qatar, conducted by the Ministry of Public Health from January 1, 2017, to December 31, 2019. Individuals attending 28 primary healthcare centers, the Gastroenterology Department at Hamad Medical Corporation (HMC), and those receiving home care were invited to voluntarily provide a blood sample for HCV Ab testing. Participation was entirely voluntary and not part of any mandatory requirement. Participants testing positive for HCV Ab were referred to HMC, the national provider of public healthcare, for additional testing, care, and treatment. Socio-demographic data collected included age, sex, and nationality. Researchers were granted access to the testing database for data analysis.

The testing protocol employed the Elecsys Anti-HCVII electrochemiluminescence immunoassay on the Cobas e 801 analyzer (Roche Diagnostics International Ltd, Rotkreuz, Switzerland). Results were interpreted according to the manufacturer’s instructions^25^. The assay has a reported sensitivity of 100% and a specificity of 99.85% (95% CI 99.73–99.93%)^25^. All methods were performed in accordance with relevant guidelines and regulations.

### Oversight

The institutional review boards at Weill Cornell Medicine–Qatar (IRB#: 24 -00006; Date of approval: March 5, 2024) and the Primary Health Care Corporation (Ref: PHCC/DCR/2019/07/016; Date of approval: November 10, 2019) approved this retrospective study with a waiver of informed consent. This cross-sectional study followed the Strengthening the Reporting of Observational Studies in Epidemiology (STROBE) guidelines (Table S1).

### Statistical analysis

Characteristics of study participants were described using frequency distributions and measures of central tendency. HCV Ab prevalence in the testing sample was calculated as the ratio of the number of individuals testing positive to the total number of individuals tested. In this computation, only the first test result for each individual was considered.

In addition to calculating the overall HCV Ab prevalence in the testing sample, HCV Ab prevalence was estimated by nationality for nationalities that contributed at least 100 participants to the study sample. The regional classification for each nationality was guided by the WHO and United Nations Geoscheme^26,27^.

HCV Ab prevalence in the resident population of Qatar was determined by applying probability weights to the testing sample. These weights were used to account for the unequal selection of participants based on age, sex, and nationality, ensuring that the estimated prevalence accurately represented the demographic composition of Qatar’s resident population from which the testing sample was drawn. Probability weights were calculated based on the distribution of Qatar’s resident population by age, sex, and nationality, using data from Qatar’s national, federated database for COVID-19 laboratory testing, which covers virtually the entire resident population of the country^18,19,28,29^. Additional details about this national database have been previously reported^18,19,28,29^.

Specimens with indeterminate test results were excluded from both the numerator and denominator in all analyses. Chi-square tests and both univariable and multivariable logistic regression analyses were used to explore associations with HCV Ab positivity. Covariates with a *p*-value of ≤ 0.2 in the weighted univariable regression analysis were considered eligible for inclusion in the weighted multivariable regression model. A *p*-value < 0.05 in the weighted multivariable analysis was deemed indicative of a statistically significant association. The results of the analyses were presented as odds ratios (ORs) and adjusted ORs (AORs), along with their respective 95% confidence intervals (CIs) and corresponding *p*-values. The statistical analyses were performed using Stata/SE version 16.1 (Stata Corporation, College Station, TX, USA).

## Results

### Study sample

A total of 81,615 individuals, constituting approximately 3% of Qatar’s resident population, participated in this testing campaign. Table 1 shows the characteristics of the participants. The median age was 31.0 years, with 79.1% being females. The participants represented a diverse array of nationalities, encompassing a total of 137 different nationalities. Predominant among the attendees were Qataris (28.9%), Egyptians (14.2%), Indians (12.8%), and Pakistanis (6.3%).

**Table 1:** Characteristics of participants in the National awareness and surveillance campaign for HCV infection in Qatar, conducted from January 1, 2017, to December 31, 2019.

Characteristics	*N* (%) *N* = 81,615
Median age (IQR)—years	31.0 (26.0–37.0)
Age (years)	
0–9	365 (0.5)
10–19	3791 (4.6)
20–29	30,600 (37.5)
30–39	30,413 (37.3)
40–49	9483 (11.6)
50–59	4823 (5.9)
60–69	1713 (2.1)
70+	427 (0.5)
Sex	
Female	64,541 (79.1)
Male	17,074 (20.9)
Nationality*	
Bangladeshi	1773 (2.2)
Egyptian	11,608 (14.2)
Filipino	4506 (5.5)
Indian	10,418 (12.8)
Iranian	935 (1.2)
Jordanian	3155 (3.9)
Pakistani	5101 (6.3)
Palestinian	1220 (1.5)
Qatari	23,592 (28.9)
Saudi	1308 (1.6)
Sri Lankan	1025 (1.3)
Sudanese	2822 (3.5)
Syrian	2891 (3.5)
Tunisian	1351 (1.7)
Yemeni	2314 (2.8)
Other^†^	7596 (9.3)
Year of first HCV Ab test^‡^	
2017	27,100 (33.2)
2018	30,276 (37.1)
2019	24,239 (29.7)
Result	
Negative	80,299 (98.4)
Positive	1149 (1.4)
Indeterminate	167 (0.2)

*Ab* antibody, *HCV* hepatitis C virus, *IQR* interquartile range.

*Nationalities were reported explicitly if their representation in the tested sample was equal to or greater than 1%.

^†^Other nationalities are: Afghan, Albanian, Algerian, American, Antiguan, Argentinean, Armenian, Australian, Austrian, Azerbaijani, Bahraini, Belarusian, Belgian, Belizean, Beninese, Bhutanese, Bolivian, Bosnian, Brazilian, British, Bruneian, Bulgarian, Burkinabe, Burmese, Cameroonian, Canadian, Chadian, Chilean, Chinese, Colombian, Comoran, Congolese, Croatian, Cuban, Cypriot, Czech, Danish, Djiboutian, Dominican, Ecuadorean, Emirati, Eritrean, Estonian, Ethiopian, Fijian, Finnish, French, Gambian, Georgian, German, Ghanaian, Greek, Guinean, Hungarian, Indonesian, Iraqi, Irish, Italian, Japanese, Kazakhs, Kenyan, Kuwaiti, Kyrgystani, Latvian, Lebanese, Libyan, Lithuanian, Luxembourger, Macedonian, Malaysian, Malian, Mauritanian, Mauritian, Mexican, Moldovan, Monacan, Montenegran, Moroccan, Mozambican, Nepalese, Netherlands, New Zealander, Nigerian, Nigerien, Norwegian, Omani, Peruvian, Polish, Portuguese, Kosovan, Romanian, Russian, Rwandan, Saint Kitts and Nevis, Salvadoran, Senegalese, Serbian, Seychellois, Singaporean, Slovak, Slovenian, Somali, South African, South Korean, Spanish, Swedish, Swiss, Tajik, Tanzanian, Thai, Togolese, Trinidadian or Tobagonian, Turkish, Ugandan, Ukrainian, Uruguayan, Uzbekistani, Venezuelan, Vietnamese, Zimbabwean, and Unknown.

^‡^The year of the HCV test corresponds to the year of the first test result for each individual considered.

### Sample and population HCV Ab prevalence

Out of the 81,615 individuals who underwent HCV Ab testing, 1149 tested positive on their first test, 80,299 tested negative, and 167 had indeterminate test results (Table 1). The estimated HCV Ab prevalence in the study sample was 1.4% (95% CI 1.3–1.5%). HCV Ab prevalence by nationality and WHO region of origin is shown in Table 2. The highest prevalence was observed in the Eastern Mediterranean region at 1.8% (95% CI 1.7–1.9%).

**Table 2:** HCV Ab prevalence by nationality and WHO region of origin among the tested participants.

WHO region	Country	Total tested^*†^ (*N*)	HCV positive (*n*)	HCV negative (*n*)	HCV indeterminate (*n*)	Prevalence^‡^ in % (95% CI^‡^)
African region	Algeria	324	0	324	0	0.00 (0.00-1.13)
	Eritrea	208	0	207	1	0.00 (0.00-1.76)
	Ethiopia	571	2	569	0	0.35 (0.04–1.26)
	Kenya	129	1	127	1	0.78 (0.02–4.24)
	Mauritania	134	0	134	0	0.00 (0.00-2.72)
	Nigeria	389	1	387	1	0.26 (0.00-1.42)
	Total^§^	1981	4	1971	6	0.20 (0.06–5.20)
Eastern Mediterranean region	Afghanistan	104	2	102	0	1.92 (0.23–6.77)
	Bahrain	459	2	457	0	0.44 (0.05–1.57)
	Egypt	11,608	711	10,851	46	6.13 (5.70–6.58)
	Iran	935	5	929	1	0.53 (0.17–1.24)
	Iraq	288	2	286	0	0.69 (0.08–2.49)
	Jordan	3155	11	3140	4	0.35 (0.17–0.62)
	Lebanon	640	2	636	2	0.31 (0.03–1.12)
	Morocco	585	1	584	0	0.17 (0.00-0.95)
	Oman	502	5	496	1	1.00 (0.32–2.31)
	Pakistan	5101	110	4976	15	2.16 (1.78–2.59)
	Palestine	1220	7	1212	1	0.57 (0.23–1.18)
	Qatar	23,592	180	23,372	40	0.76 (0.66–0.88)
	Saudi Arabia	1308	8	1299	1	0.61 (0.26–1.20)
	Somalia	277	2	275	0	0.72 (0.09–2.58)
	Sudan	2822	13	2800	9	0.46 (0.25–0.79)
	Syria	2891	14	2875	2	0.48 (0.27–0.81)
	Tunisia	1351	1	1347	3	0.07 (0.00-0.41)
	United Arab Emirates	162	0	162	0	0.00 (0.00-2.25)
	Yemen	2314	8	2299	7	0.35 (0.15–0.68)^¶^
	Total^§^	59,468	1084	58,251	133	1.82 (1.71–1.93)
European region	Turkey	120	0	120	0	0.00 (0.00-3.03)
	United Kingdom	523	4	518	1	0.76 (0.21–1.95)
	Total^§^	1232	11	1218	3	0.89 (0.44–1.59)
Region of the Americas	Canada	146	2	144	0	1.37 (0.17–4.86)
	United States of America	222	4	217	1	1.80 (0.49–4.55)
	Total^§^	454	6	447	1	1.32 (0.49–2.85)
South-East Asia region	Bangladesh	1773	6	1766	1	0.34 (0.12–0.74)
	India	10,418	25	10,377	16	0.24 (0.16–0.35)
	Indonesia	144	0	144	0	0.00 (0.00-2.52)
	Nepal	377	0	376	1	0.00 (0.00-0.97)
	Sri Lanka	1025	2	1023	0	0.20 (0.00-0.70)
	Total^§^	13,785	33	13,734	18	0.24 (0.16–0.34)
Western Pacific region	Philippines	4506	11	4490	5	0.24 (0.12–0.43)
	Total^§^	4693	11	4676	6	0.23 (0.12–0.42)

*Ab* antibody, *CI* confidence interval, *HCV* hepatitis C virus, *WHO* World Health Organization.

^*^Countries were explicitly reported if their representation in the tested sample was equal to or greater than 100 participants.

^†^Excludes two participants with unknown nationality.

^‡^The reported prevalence is the sample (unweighted) prevalence. The first antibody test for each individual was considered in the calculation of prevalence.

^§^The regional total comprises all countries within the respective region, including those with tested samples of fewer than 100 participants.

^¶^Although HCV Ab prevalence among Yemenis appears to be low, the Ab-positive cases were predominantly young, leading to a strong association between Yemeni nationality and HCV Ab positivity observed in Table 3.

After applying probability weights to the testing sample, the estimated HCV Ab prevalence in the resident population of Qatar was 1.4% (95% CI 1.2–1.7%).

### Associations with HCV Ab positivity

Table 3 provides the ORs and AORs describing the associations with being HCV Ab positive. HCV Ab positivity was significantly associated with age, showing higher AORs as age increased, indicating an increased likelihood of testing positive. Compared to individuals aged 20–29 years, the AOR was 1.83 (95% CI 0.79–4.22) for those aged 30–39, 2.78 (95% CI 1.24–6.25) for those aged 40–49, 4.86 (95% CI 2.16–10.92) for those aged 50–59, 6.04 (95% CI 2.65–13.77) for those aged 60–69, and 11.75 (95% CI 4.29–32.18) for individuals over 70 years of age.

**Table 3:** Associations with HCV antibody positivity in Qatar.

Characteristics	Total tested	HCV antibody-positive		Univariable regression analysis				Multivariable regression analysis^*^	
	N^†^	n (%^‡^**)**	*p*-value	OR^§^ (95% CI^§^**)**	*p*-value	F test* p*-value^¶^	R^**2**^ (%)	AOR^§^ (95% CI^§^**)**	*p*-value**
Age (years)						< 0.001	12.93		
20–29	30,572	133 (0.2)	< 0.001	1.00				1.00	
0–9	365	8 (0.0)		1.39 (0.45–4.33)	0.566			0.97 (0.32–2.97)	0.954
10–19	3,786	13 (0.0)		0.57 (0.20–1.60)	0.283			0.39 (0.14–1.10)	0.076
30–39	30,375	360 (0.4)		1.82 (0.81–4.08)	0.144			1.83 (0.79–4.22)	0.157
40–49	9,463	248 (0.3)		2.83 (1.29–6.20)	0.009			2.78 (1.24–6.25)	0.013
50–59	4,806	279 (0.2)		4.86 (2.22–10.62)	< 0.001			4.86 (2.16–10.92)	< 0.001
60–69	1,705	119 (0.1)		6.21 (2.75–13.99)	< 0.001			6.04 (2.65–13.77)	< 0.001
70+	425	34 (0.0)		11.70 (4.45–30.81)	< 0.001			11.75 (4.29–32.18)	< 0.001
Sex									
Female	64,458	520 (0.3)	< 0.001	1.00		< 0.001	21.18	1.00	
Male	17,039	674 (1.2)		1.96 (1.47–2.61)	< 0.001			1.97 (1.47–2.65)	< 0.001
Nationality^††^									
Qatari	23,565	193 (0.0)	< 0.001	1.00		< 0.001	49.02	1.00	
Bangladeshi	1,773	6 (0.0)		0.29 (0.12–0.71)	0.007			0.21 (0.08–0.52)	0.001
Egyptian	11,574	726 (0.4)		12.24 (9.80-15.28)	< 0.001			11.40 (8.83–14.73)	< 0.001
Filipino	4,502	11 (0.0)		0.72 (0.32–1.60)	0.416			0.71 (0.31–1.61)	0.417
Indian	10,407	26 (0.2)		0.89 (0.47–1.68)	0.716			0.67 (0.36–1.26)	0.217
Iranian	934	6 (0.0)		2.10 (0.85–5.25)	0.110			1.33 (0.53–3.37)	0.542
Jordanian	3,152	12 (0.0)		1.27 (0.47–3.39)	0.638			1.20 (0.45–3.21)	0.722
Pakistani	5,088	117 (0.3)		8.20 (5.55–12.10)	< 0.001			7.06 (4.69–10.63)	< 0.001
Palestinian	1,219	7 (0.0)		0.87 (0.34–2.25)	0.778			0.71 (0.27–1.85)	0.485
Saudi	1,307	9 (0.0)		1.76 (0.57–5.45)	0.325			1.59 (0.52–4.84)	0.413
Sri Lankan	1,025	2 (0.0)		0.46 (0.07–3.13)	0.425			0.34 (0.05–2.32)	0.272
Sudanese	2,815	13 (0.0)		0.57 (0.24–1.37)	0.208			0.47 (0.20–1.13)	0.091
Syrian	2,891	15 (0.0)		1.76 (0.85–3.65)	0.126			1.60 (0.76–3.37)	0.211
Tunisian	1,350	1 (0.0)		0.03 (0.00-0.24)	0.001			0.03 (0.00-0.24)	0.001
Yemeni	2,309	10 (0.0)		5.39 (1.18–24.68)	0.030			5.83 (1.19–28.42)	0.029
Other^‡‡^	7,586	40 (0.3)		1.67 (1.00-2.78)	0.050			1.36 (0.82–2.24)	0.229
Year of the first HCV test^§§^									
2017	26,946	373 (0.5)	0.056	1.00		0.780	0.25	–	–
2018	30,276	422 (0.5)		0.87 (0.57–1.32)	0.505			–	–
2019	24,275	393 (0.5)		0.98 (0.68–1.41)	0.906			–	–

*AOR* adjusted odds ratio, *CI* confidence interval, *HCV* hepatitis C virus, *OR* odds ratio.

*The multivariable model explained 53.1% of the variability in HCV antibody positivity.

^†^The specimens with indeterminate test results were excluded from both the numerator and denominator.

^‡^The percentage of positive cases out of those tested was weighted by age, sex, and nationality.

^§^Estimates were weighted by age, sex, and nationality.

^¶^Covariates with *p*-value ≤ 0.2 in the univariable analysis were included in the multivariable analysis.

^**^Covariates with *p*-value < 0.05 in the multivariable analysis were considered as showing strong evidence for an association with HCV antibody positivity.

^††^Nationalities were reported explicitly if their representation in the tested sample was equal to or greater than 1%.

^‡‡^Other nationalities are: Afghan, Albanian, Algerian, American, Antiguan, Argentinean, Armenian, Australian, Austrian, Azerbaijani, Bahraini, Belarusian, Belgian, Belizean, Beninese, Bhutanese, Bolivian, Bosnian, Brazilian, British, Bruneian, Bulgarian, Burkinabe, Burmese, Cameroonian, Canadian, Chadian, Chilean, Chinese, Colombian, Comoran, Congolese, Croatian, Cuban, Cypriot, Czech, Danish, Djiboutian, Dominican, Ecuadorean, Emirati, Eritrean, Estonian, Ethiopian, Fijian, Finnish, French, Gambian, Georgian, German, Ghanaian, Greek, Guinean, Hungarian, Indonesian, Iraqi, Irish, Italian, Japanese, Kazakhs, Kenyan, Kuwaiti, Kyrgystani, Latvian, Lebanese, Libyan, Lithuanian, Luxembourger, Macedonian, Malaysian, Malian, Mauritanian, Mauritian, Mexican, Moldovan, Monacan, Montenegran, Moroccan, Mozambican, Nepalese, Netherlands, New Zealander, Nigerian, Norwegian, Omani, Peruvian, Polish, Portuguese, Kosovan, Romanian, Russian, Rwandan, Saint Kitts and Nevis, Salvadoran, Senegalese, Serbian, Seychellois, Singaporean, Slovak, Slovenian, Somali, South African, South Korean, Spanish, Swedish, Swiss, Tajik, Tanzanian, Thai, Togolese, Trinidadian or Tobagonian, Turkish, Ugandan, Ukrainian, Uruguayan, Uzbekistani, Venezuelan, Vietnamese, Zimbabwean, and Unknown.

^§§^The year of the HCV test corresponds to the year of the first antibody-positive test for individuals with at least one antibody-positive test and to the year of the first negative test for individuals who had only antibody-negative tests.

For most nationalities, there were no statistically significant differences in HCV Ab positivity compared to Qataris (Table 3). However, for certain nationalities, large differences were observed. Tunisians and Bangladeshis had 0.03-fold (95% CI 0.00–0.24) and 0.21-fold (95% CI 0.08–0.52) lower odds of being HCV Ab positive, respectively, compared to Qataris. Conversely, Yemenis, Pakistanis, and Egyptians had 5.83-fold (95% CI 1.19–28.42), 7.06-fold (95% CI 4.69–10.63), and 11.41-fold (95% CI 8.83–14.73) higher odds of being HCV Ab positive, respectively, compared to Qataris.

Males had a 1.97-fold (95% CI 1.47–2.65) higher odds of being HCV Ab positive compared to females (Table 3). There were no differences in HCV Ab positivity observed based on the year of the test.

## Discussion

The estimated HCV Ab prevalence in the resident population of Qatar is 1.4% (95% CI 1.2–1.6%), surpassing the previous estimate derived from a meta-analysis conducted a few years ago, which pooled existing HCV Ab prevalence data and indicated a prevalence of 0.51% (95% CI 0.43–0.59)^30^, as well as the 0.8% prevalence observed among craft and manual workers in Qatar^20^. This higher prevalence highlights the considerable progress needed in Qatar to achieve the WHO elimination target by 2030.

However, this overall HCV Ab prevalence masks significant disparities based on nationality. Notably, Egyptians and Pakistanis exhibited higher HCV Ab positivity compared to Qataris. This is consistent with the documented high levels of HCV Ab prevalence in Egypt^12,31–33^ and Pakistan^34–37^, countries known to have some of the highest levels globally^20,23,24^. Similar findings were also observed in a study of craft and manual workers in Qatar^20^. HCV Ab positivity appeared also relatively high among nationals from few countries with limited HCV data availability, such as Yemen^21^. This highlights the need for further studies to measure the prevalence of this infection in these countries where substantial prevalence levels may remain poorly documented due to limited viral hepatitis programming and HCV treatment efforts.

HCV Ab positivity displayed a pronounced age dependency, with substantially higher levels observed among older individuals. This trend not only reflects the increasing likelihood of HCV exposure with more years of life, but also suggests a strong cohort effect. Many HCV infections may have been acquired before widespread improvements in blood supply screening, injection safety, and infection control measures were implemented globally over the past 3 decades following the discovery of this infection^13,14,38^. Similar cohort effects have been observed across countries in the MENA region^21,22,30–33,35,36,39–42^, indicating that many HCV exposures were linked to specific healthcare procedures such as blood transfusions, surgeries, and hemodialysis^16,43–45^. Additionally, being male was associated with a higher prevalence, consistent with certain exposures such as injecting drug use^46,47^ and parenteral antischistosomal therapy specifically in Egypt^31,41^, which are more common among males than females.

These findings underscore the need for targeted screening^48^, treatment expansion, and harm reduction strategies. While highly effective pan-genotypic therapies achieve cure rates exceeding 95% ^9,10^, ensuring their broad accessibility through public healthcare services remains essential. Populations from high-prevalence countries, often employed in labor-intensive sectors in Qatar, may face barriers to testing and treatment, necessitating targeted outreach. Culturally tailored awareness campaigns and streamlined referral pathways are critical for early diagnosis and treatment initiation.

Integrating targeted HCV screening into routine healthcare, particularly in primary care, could enhance case detection. Qatar’s extensive healthcare infrastructure provides a key opportunity to reach at-risk individuals by optimizing case-finding strategies through electronic medical record flagging, physician education, and community-based initiatives to improve treatment uptake. Sustained investment in HCV programming, alongside innovative approaches such as point-of-care testing and decentralized treatment models, is vital to reducing the infection burden and accelerating progress toward elimination.

This study has limitations. Firstly, only basic demographic data were collected from tested individuals, limiting the ability to explore associations with other potential risk factors such as occupation, education, and specific exposure histories related to HCV. Secondly, the estimated HCV Ab prevalence may not reflect the true prevalence in the broader population, as the testing sample was drawn from individuals attending healthcare centers. This approach may underrepresent marginalized or hard-to-reach populations, such as people who inject drugs^46,47,49^. However, the inclusion of multiple testing sites across Qatar likely captured geographic and population variations in prevalence, enhancing the representativeness of the findings.

Thirdly, as this study relied on voluntary testing, it is plausible that those who consented to be tested are individuals with risk factors for HCV infection or are aware of its significance based on their home country context. This is evident in the higher proportion of Egyptians and Pakistanis in the testing sample compared to their demographic representation in the resident population of Qatar^18,19,28,29^. Consequently, there may be an overestimation of HCV Ab prevalence. However, the study used probability weights to adjust for the unequal selection of participants based on age, sex, and nationality, and had a large sample size, which may have mitigated the impact of this bias.

Fourthly, although the study provided estimates for HCV Ab prevalence across various nationalities, it is essential to interpret these prevalence levels cautiously. They serve more as indicators of potential prevalence in the individuals’ home countries rather than definitive estimates. This is because the prevalence was calculated based on a select sample of individuals who arrived in Qatar from these countries.

Fifthly, the study utilized HCV Ab testing, but we lacked access to the clinical data of Ab-positive participants who were linked to care to confirm the results through confirmatory testing. Data on polymerase chain reaction testing to confirm current infection were also unavailable, precluding the assessment of the prevalence of current infection in the population and the viremic rate, which represents the proportion of individuals who tested Ab positive and have not cleared or treated their chronic infection^50,51^. Nevertheless, this study was primarily designed as a retrospective sero-surveillance analysis of existing data to inform national HCV response strategies rather than to provide a state-of-the-art prospective epidemiological characterization of HCV infection in Qatar.

Lastly, while the study employed a widely used assay with near-perfect specificity (99.85%)^25^, the possibility of false-positive results cannot be entirely ruled out, particularly in real-world testing conditions. Such misclassifications are more likely to influence prevalence estimates in populations with low infection rates, as even a small number of false positives could lead to overestimations. This consideration is particularly relevant for nationalities with low HCV prevalence, where the impact of potential misclassification may be proportionally greater.

This study has strengths. Firstly, the testing sample is large, encompassing approximately 3% of Qatar’s resident population. Such a large sample size enabled the generation of various analyses and inferences, as well as precise estimations of outcomes. Secondly, the considerable diversity within the testing sample, comprising individuals from a wide range of countries, facilitated the estimation of HCV Ab prevalence across various nationalities. This diversity provided insights into the potential prevalence of HCV in home countries, particularly in nations where data on HCV prevalence remain limited.

## Conclusions

HCV Ab prevalence in the resident population of Qatar was estimated at 1.4%, highlighting the progress required to reach the WHO elimination target by 2030. However, this overall prevalence masked significant disparities based on nationality within the population, with notably high positivity among Egyptians, Pakistanis, and Yemenis. Moreover, HCV Ab prevalence exhibited a pronounced age dependency, suggesting a cohort effect likely linked to past healthcare exposures. These findings emphasize the importance of targeted interventions, prevention and harm reduction strategies, and expanded testing and treatment programs to prevent HCV transmission, reduce associated disease burden, and advance progress toward the 2030 elimination target.

## Electronic supplementary material

Below is the link to the electronic supplementary material.

Supplementary Material 1

## Data Availability

The dataset of this study is a property of the Qatar Ministry of Public Health that was provided to the researchers through a restricted-access agreement that prevents sharing the dataset with a third party or publicly. The data are available under restricted access for preservation of confidentiality of patient data. Access can be obtained through a direct application for data access to Her Excellency the Minister of Public Health (https:// www.moph.gov.qa/english/OurServices/eservices/Pages/Governmental-HealthCommunication-Center.aspx). The raw data are protected and are not available due to data privacy laws. Aggregate data are available within the paper.
